# Attachment to Mother and Father at Transition to Middle Childhood

**DOI:** 10.1007/s10826-016-0602-7

**Published:** 2016-11-14

**Authors:** Simona Di Folco, Serena Messina, Giulio Cesare Zavattini, Elia Psouni

**Affiliations:** 1 Department of Dynamic and Clinical Psychology, University of Rome, Sapienza, Italy; 2 Department of Educational Psychology, University of Texas at Austin, Austin, TX USA; 3 Department of Psychology, Lund University, Lund, Sweden

**Keywords:** Middle childhood, Secure base script, Attachment to father, Attachment to mother, Generalized attachment representations

## Abstract

The present study investigated concordance between representations of attachment to mother and attachment to father, and convergence between two narrative-based methods addressing these representations in middle childhood: the *Manchester Child Attachment Story Task* (MCAST) and the *Secure Base Script Test* (SBST). One hundred and twenty 6-year-old children were assessed by separate administrations of the MCAST for mother and father, respectively, and results showed concordance of representations of attachment to mother and attachment to father at age 6.5 years. 75 children were additionally tested about 12 months later, with the SBST, which assesses scripted knowledge of secure base (and safe haven), not differentiating between mother and father attachment relationships. Concerning attachment to father, dichotomous classifications (MCAST) and a continuous dimension capturing scripted secure base knowledge (MCAST) converged with secure base scriptedness (SBST), yet we could not show the same pattern of convergence concerning attachment to mother. Results suggest some convergence between the two narrative methods of assessment of secure base script but also highlight complications when using the MCAST for measuring attachment to father in middle childhood.

## Introduction

Attachment theory regards the caregiver both as secure base when fostering the child’s exploration and as safe haven in times of need (Ainsworth et al. 1978; Bowlby 1969/1982, 1973). Experiences with a sensitive and responsive caregiver promote knowledge, on the child’s side, that the caregiver will be available when needed (Waters and Cummings 2000). As cognitive, linguistic and social skills mature, representations of sensorimotor experiences of early parent-child interactions become internalized into working models (IWMs: Bowlby 1973; Bretherton 1991), to allow the child to mentally “bring close” the caregiver whose physical proximity cannot be guaranteed at all times, by creating a mental representation of the relationship with him/her, thereby moderating distress from separation and facilitating exploration in the caregiver’s absence (Bowlby 1973, 1980). IWMs serve also as guide for behavior and affect regulation in future relationships (Bretherton and Munholland 2016; Main et al. 1985). These emerging representations, starting at preschool and being remarkably evident in middle childhood, denote a developmental shift in the attachment system, from behavioral to more representational in school age. There is evidence that early attachment representations are relatively stable over time and that adult attachment representations are congruent with attachment formed in early childhood (Fraley 2002; Grossmann et al. 2008).

However, less is known about how different components and facets of attachment representations evolve through development. For instance, attachment representations concerning mother and father are relationship-specific but there is evidence of concordance in their quality already during early childhood (Diener et al. 2008; Fox et al. 1991; Monteiro et al. 2008; Steele et al. 1996; Van IJzendoorn and De Wolff 1997; Veríssimo et al. 2011). One question concerns the extent to which mother-related and father-related attachment representations merge into a generalized representation by the end of early childhood, as a prerequisite for the unitary working model of attachment that allegedly emerges in late childhood and adolescence (Dykas et al. 2006; Waters et al. 2015). Attachment relationships to mother and father may not contribute similarly in shaping children’s generalized representations. For instance, while there is evidence that fathers play a more important role in teaching emotionally and socially appropriate behaviors with peers, the attachment relationship to mother seems more relevant for the development of emotional understanding and the integration of positive and negative feelings in children’s autobiographical narratives (Steele and Steele 2005). However, coding attachment categorically restricts the question of relationships between attachment representations to mother and father, respectively, into whether they convergence or not. Considering how attachment models regarding mother and father may vary from, or complement, one another may be a more useful approach for furthering our understanding of each parent’s unique contribution to the child’s attachment development toward a generalized representation (see also Miljkovitch et al. 2015).

Middle childhood is a crucial period for child development, characterized by remarkable changes in social cognition and emotion regulation. As children spend more time away from caregivers and interact with wide social networks, the goal of the attachment system changes from caregiver proximity to caregiver availability (Ainsworth 1990; Mayseless 2005). Attachment relationships with parents can now be viewed as goal directed partnerships, as children can understand their parents’ desires and decisions, negotiate their own attachment-related plans (Kerns et al. 2006) and actively monitor and influence parental availability accordingly. Attachment development in middle childhood is also marked by a shift toward integrated representations of different attachment relationships within the family (Bretherton and Munholland 2016), while expanded social interactions outside the family call for further generalization of these representations (Bowlby 1980), to allow children to explore these new situations with the guarantee that their parents will provide safety but also support. Consistently, while recent evidence suggests that secure base script knowledge by adolescence is generalized across relationships and continuously distributed (Waters et al. 2015), cognitive schemas related to attachment are thought to still be under development in middle childhood, benefiting from the influence of these new social experiences and novel attachment relationships (Bosmans and Kerns 2015). Several questions remain unanswered concerning the specifics of these processes of attachment development in middle childhood.

Kerns and Seibert (2011) pointed out the diversity in methods for operationalizing and measuring attachment in middle childhood. While attachment assessment in preschool years relies on observation of behaviors during separation and reunion procedures (Main and Cassidy 1988), toward the end of early childhood children are less sensitive to brief separations from parents, as their attachment representations become more elaborate because of strengthened verbal and memory skills (Messina and Zavattini 2014). Indeed, verbal and memory skills play an important role in the construction and reconstruction of attachment representations (Waters et al. 1998), which is also supported by the association between attachment security and children’s intellectual functioning in general (Jacobsen et al. 1994; Jacobsen and Hofmann 1997) and verbal ability in specific (Atkinson et al. 1999; O’Connor and McCartney 2007; Stievenart et al. 2010; Van IJzendoorn et al. 1995; Van IJzendoorn and Van Vliet-Visser 1988). Thus, the more advanced the child’s verbal abilities, the better the ability to represent attachment relationships.

Consistently, narrative measures based on story completion have been proposed to assess children’s attachment representations in middle childhood, reflecting the centrality of language as means of communicating emotions and needs and negotiating goals (Waters and Cummings 2000) and the representational structure of memories important for succeeding in socioemotional goals in everyday interactions (Fivush et al. 1996; Hudson 1990; Welch-Ross 1995). The main premise of story completion tasks is that children actualize in their narratives their internal representations of attachment relationships, expressed as relational themes, defences, and coping strategies (Bretherton et al. 1990; Cassidy 1988; Psouni and Apetroaia 2014). Story completion tasks may at the same time be regarded as measurements of the child’s scripted secure base knowledge in situations involving distress (Psouni and Apetroaia 2014; Waters and Waters 2006). The core of this scripted knowledge includes the following elements: (1) caregiver supporting the child’s exploration, remaining available, and responsive; (2) when the child encounters a difficulty, either child seeking caregiver or caregiver going to child; (3) difficulty being dealt with; (4) proximity and contact with caregiver effectively comforting the child; and (5) caregiver facilitating a return to exploration or a transition to another activity (Psouni and Apetroaia 2014; see also Waters et al. 1998).

Among methods employing story completion, the well-validated *Manchester Child Attachment Story Task* (MCAST: Goldwyn et al. 2000; Green et al. 2000) explores attachment representations in 5- to 7-year-old children, providing both an attachment classification and continuous scores on qualities such as engagement and arousal of the child during participation in the task, attachment-related and caregiving behaviors, narrative coherence, and disorganization. The MCAST is less suitable for use with children older than 7 (Green et al. 2000) as the doll-play setting may be inappropriate for engaging older children (although Barone et al. (2009) did use it with children as old as 8 years). Another validated method employing story completion, the *Secure Base Script Test* (SBST: Psouni and Apetroaia 2014) for assessing scripted secure base knowledge, is based on word outlines that prompt attachment-related narratives similar to those enacted by the dolls in the MCAST. The test was designed for use with children aged 7-years or older (Psouni and Apetroaia 2014; Psouni et al. 2015) and the continuous, single variable score from it, reflecting scripted secure base knowledge, converges with evidence of secure base availability and responsiveness of caregivers as assessed by the *Friends and Family Interview* (Steele and Steele 2005), an attachment interview appropriate for use with children in this age group (Psouni and Apetroaia 2014). Unlike the MCAST, which assesses parent-specific attachment relationships, the SBST assesses scripted attachment knowledge considering mother and father together. As most studies in middle childhood have used a single measure of attachment, the exact ways in which different measures are related to each other in middle childhood is unknown, but the strong similarity in methodology and attachment themes addressed suggests that the two methods should converge in assessing attachment security in general, and scripted secure base knowledge in specific.

During transition to middle childhood, the exploratory—rather than the caregiving—system becomes increasingly important for the child’s further development. It has been suggested that the child’s explorative behavior may be particularly encouraged within the attachment relationship with the father (Bretherton 2010; Kerns et al. 2015), and evidence suggests that, indeed, fathers expose children to more challenging games and activities than mothers do (Cabrera et al. 2014; Paquette and Dumont 2013). Furthermore, relationships to peers become more important in middle childhood and studies have highlighted the importance of attachment to father for positive relationships with peers (Booth-Laforce et al. 2006; Diener et al 2008; Freitag et al. 1999; Grossmann et al. 2002; Steele and Steele 2005; Veríssimo et al. 2011). Thus, the attachment relationship to father may become particularly salient during middle childhood. Yet, the few studies that included a father character in story stems have focused on adoptive families (Barone and Lionetti 2011), single parent (Bernier and Miljkovitch 2009; Miljkovitch et al. 2012), and post-divorce families (Page and Bretherton 2001), while only one study concerned attachment representations to mother and father, respectively, in children living with two parents (Portu-Zapirain 2013).

The main aim of this work was to investigate representations of attachment at a time around the transition from early to middle childhood. The MCAST was administered twice within a period of 3 months, using story stems including a mother character at one administration and a father character at the other administration and obtaining separate classifications of attachment to mother and father, respectively, replicating procedures used by Barone and Lionetti (2011). On the basis of previous literature concerning concordance of attachment representations in early middle childhood (Booth-LaForce et al. 2006; Diener et al. 2008; Kochanska and Kim 2013), we expected concordance of 6-to-7-year-old children’s attachment representations to mother and father, respectively. A second aim was to assess convergence between dichotomous (secure/insecure) classifications obtained with the MCAST and SBST. Thus, children were tested a third time within a year from the previous test points, with the SBST. As the categorical approach often used to describe attachment (Gloger-Tippelt et al. 2002; Moss et al. 2005) has been criticized for not allowing the study of variations among individuals within categories while analysis of continuous subscales reflecting features of the attachment representation may capture such variations (Stievenart et al. 2014; Cummings 2003), we also applied a continuous approach in addition to the categorical approach, exploring the link between children’s scripted secure base knowledge as measured by continuous scales in the MCAST and by the SBST. This approach is in line with recent empirical evidence that the underlined structure of IWMs in adulthood may be better understood as a continuous, rather than a taxonomic, model (Fraley and Roisman 2014). Relying on continuous measures of attachment allows us to also assess the degree of overlap across models of attachment relationships to mother and father, respectively, rather than only consider concordance, or not (Fox et al. 1991; Furman and Simon 2004). As previous studies indicate reciprocal relations between pre-schoolers’ verbal IQ and attachment representations (Stievenart et al. 2010; Van IJzendoorn et al. 1995), verbal IQ was controlled for.

## Method

### Participants

In total, 120 first grade children (61 male and 59 female; *M*_*Age*_ = 76.4 months or 6 years and 4-months-old, SD = 4.06) were assessed at age 6 twice, with 3 months in between sessions, and 75 children were also assessed after about 12 months (62.5 % of the initial sample: 32 male and 42 female; *M*_*Age*_ = 91.1 months or 7 years and 7-months-old, SD = 11.43). Children were Caucasian, recruited in the city of Rome and nearby, from five primary public schools. They lived in two-parent families, had no special teaching support and had not been referred to public local health services for psychopathological symptoms. Mothers’ mean age was 38.7 years (SD = 3.90) and fathers’ 43.0 years (SD = 5.42). Regarding mothers, 75.5 % had an education corresponding to 7–12 years’ full time studies, 24.5 % had qualifications corresponding over 15 years’ full time study. Similarly, 83.3 % of the fathers had an education corresponding to 7–12 years’ full time studies, while 16.7 % had 15 or more years’ fulltime study. Concerning mothers, 58.2 % worked full-time, 14.5 % were employed part-time while 27.3 % were not employed at the time of the study. Among fathers, 94.4 % worked full-time while 5.6 % were not employed at the time of the study. Family average incomes were €30,000 (SD = 10,800), above average income for a family living in Northern Italy (24,600 Euro: ISTAT report 10 December 2012).

Of the 120 participating children, 3 refused to participate at test-session 2, and an additional 42 (37.5 %) were lost to attrition at test-session 3 (17 could not be contacted, 3 had moved and could not be located, 22 refused to participate). The children who participated at all three test-sessions (*N* = 75) did not differ from those who did not (*N* = 45) on income level or family type, nor were there differences in distribution of attachment between the two groups (3-way attachment to mother *χ*_*(2)*_^*2*^ = 2.54, *ns*; 3-way attachment to father *χ*_*(2)*_^*2*^ = 1.22, *ns*).

### Procedure

After approval by the Ethical Committee at the University of Rome and permission by school principals, brief meetings were arranged with parents, children, and teachers for presentation of the project and its purposes. Children were recruited subject to parental written informed consent: 154 parent pairs were asked and 120 consented to their children’s participation (78 %).

The study included 3 test-sessions for each child, over a period of approximately 15 months. Testing took place at the children’s schools, each session lasting approximately 30 min. In the first test-session, children’s attachment representations were assessed with the MCAST (for one parent) followed by the verbal subtest of the WISC-III. In the second test-session approximately 3 months later, the MCAST (with respect to the other parent) was administered. The order of MCAST-mother and MCAST-father administration was counterbalanced. In the third test-session, about 12 months later, children narrated stories according to the SBST and were again assessed with the verbal subtest of the WISC-III.

### Measures

#### Attachment assessed by the MCAST

Attachment representations were first assessed with the MCAST (Goldwyn et al. 2000; Green et al. 2000/2005; Green et al. 2000) on two occasions, focusing on relation to mother and relation to father, respectively. The MCAST is a semi-projective story completion task developed to elicit children’s narrative with respect to four attachment-related themes: *nightmare, hurt knee, tummy ache,* and *child gets lost in shopping center*. A wooden house, a mother or father doll, respectively, and a doll representing the child were used. The child was asked to complete each story introduced by the interviewer, using the dolls. Video and transcripts from each story separately were scored using 21 ordinal scales around four focal areas: (1) engagement in the task and quality of arousal (2 scales); (2) attachment-related behaviors attributed to the child in the child’s story (proximity seeking, self-care, role-reversal, and assuagement) and caregiving behaviors attributed to the parent in the story (warmth, sensitivity, disengagement, angry resistance/motivational conflict, captured by 12 scales); (3) narrative coherence and mentalizing (5 scales) and (4) disorganization related to episodic phenomena and bizarre themes (2 scales) (Green et al. 2000/2005).

The coding system results in overall attachment classifications: *Secure (B)*, where stories present children who ask for help or support, caregivers who are sensitive, supportive, neither controlling nor dismissing/withdrawing and able to provide a solution so that exploration can be restored. *Avoidant (A)* stories include a caregiver who is cold, rejecting, so the child copes with distress by self-care or displacement. There is an ineffective interaction, or lack of interaction, with the caregiver. *Ambivalent (C)* stories involve interpersonal child-caregiver relationships not effective in soothing the distress, instead increasing or feeding it, involving high levels of anger and control. Finally, *Disorganized (D)* stories are characterized by incoherence and inefficacy in dealing with distress, implying a total lack of strategy or rapid shifts between incompatible strategies. The MCAST has good inter-rater reliability, stability, and concurrent concordance with mothers’ AAI classifications (Goldwyn et al. 2000; Green et al. 2000). Findings on a non-clinical sample of children between 5- and 7-years old, comparing the MCAST with concurrent maternal attachment representation, measures of child temperament and behavior and concurrent ratings on the Separation Anxiety Test (Resnick 1993), showed that ratings of disorganized attachment on the MCAST were associated with Unresolved status on concurrent maternal AAIs, and with independent teacher ratings of classroom behavior (Goldwyn et al. 2000). These findings support convergent and criteria validity of the MCAST.

In the present study, MCAST video-recordings were coded by a reliable judge (trained by Professor Green), blind with respect to the attachment classification assigned for “the other” parent. A second reliable judge coded 20 % of videos. Inter-rater agreement was 89 % (*k* = .82) for four way (A, B, C, and D) attachment classifications. Internal consistency for the MCAST subscales in this study was Cronbach’s *α* = .72 MCAST-mother, Cronbach’s *α* = .71 MCAST-father.

Four of the original MCAST scales were thought of as capturing secure base script knowledge in children’s stories, as they fit well the description of the core elements of the secure base script (Psouni and Apetroaia 2014): *Proximity* (seeking contact and closeness by both child and caregiver when a difficulty arises), *Sensitivity* (caregiver’s physical and emotional response to the child’s distress, orientation to the child’s behavior and state of mind), *Assuagement* (degree to which child’s distress is moderated, both as a result of appropriate caregiver actions and because the child accepts the care and soothing—from the child’s and from the coder’s perspective) and *Warmth* (inferred caregiver emotional warmth in dealings with the child). The four scales were averaged to create a compound reflecting scripted secure base knowledge in the MCAST, separately for mother and father. The internal consistency for the compound was *α* = .89 for mother and *α* = .89 for father.

#### Scripted attachment knowledge assessed by the SBST

The SBST (Psouni and Apetroaia 2012, 2014) was used in the third test occasion. It assesses children’s scripted knowledge of secure base by asking them to create stories with the help of four word prompt outlines that elicit attachment-related situations, two involving secure base interactions with parents (*Math Test* and *Accident*) and two interactions with a best friend (*Troubles at school* and *Moves away*). Each storyline consists of 12 words, suggesting the main character (name matching the child’s gender) experiencing a crisis, interaction with caregivers/friend and possible resolution. The four test outlines are presented each one at a time, counterbalanced for order, and children are asked to tell the best story they can. A warm-up story (*Birthday*) is included for training. The task takes between 8 and 20 min to complete. The session is audio-recorded.

Transcribed stories are scored on a seven-point scriptedness scale (scores 1–7) reflecting amount of secure base knowledge in the child’s narrative, as defined in a scoring manual (Psouni and Apetroaia 2012). Scoring is done across stories so coders are unaware of the child’s one story when they score the child’s other stories. Narratives characterized by rich secure base content receive a score of 6 or 7, depending on degree of elaboration in the emotional interactions included, narratives containing some elements of the SBS but restricted in detail are scored as 5 and narratives with minimal but clear content indicating a secure base script are scored as 4. Narratives focusing on actions or events, not acknowledging emotional states or interactions, receive scores of 3. Stories lacking secure base content and being disconnected are scored as 2, while narratives containing odd content are scored as 1. There is evidence of high inter-rater reliability in scoring the SBST stories (.85–.95 in Psouni and Apetroaia 2014; .85 in Psouni et al. 2015). A total score is produced by averaging the scores across the four stories from each child, and high internal consistencies have been reported in previous studies (.87 in Psouni and Apetroaia 2014; .77 in Psouni et al. 2015). Construct and concurrent validity has also been demonstrated, both against the *Kerns Security Scale* (Kerns et al. 2001), a self-report measure of attachment security, and against the *Friends and Family Interview* (Steele and Steele 2005), an attachment interview appropriate for use with children and adolescents (Psouni and Apetroaia 2014).

The SBST materials and manual were translated to Italian after permission from Professor Psouni, back-translated, and scrutinized for accuracy of expression. The method was then piloted with a group of Italian children (*N* = 20, age range 84–96 months) and adjusted where necessary. All stories from the pilot were translated to English, scrutinized for content and scored by Professor Psouni and one author previously trained and tested reliable in scoring the SBST. Intra-class reliability between the method developer and the new, trained, coder was on this piloting material .85.

All SBST narratives from the present study were scored by two reliable coders, trained by Professor Psouni in March 2012. Inter-coder, intra-class reliability was .83 for *Math Test*, .84 for *Accident*, .82 for *Troubles at School* and .85 for *Moves away*. Internal consistency for the four stories was .75, somewhat lower than reported elsewhere (Psouni and Apetroaia 2014).

#### Verbal ability

The verbal subtests of the WISC-III (Wechsler 1991; Italian validation Orsini and Picone 2006) were used: Information, Similarities, Arithmetic, Vocabulary, Comprehension and Digit Span. Standardized scores (0–20) provide a measure of verbal IQ (M = 10, SD = 3), which is also highly correlated with the full-scale IQ (Wechsler 1991). Verbal ability was assessed both at the first and last test-sessions. While intelligence as psychological construct is assumed to be stable over time, it has been suggested that WISC-III subtests may be less stable than global IQ (for more information about long-term stability of the WISC-III see Canivez and Watkins 2001; for a general discussion see Moffitt et al. (1993) and repeated verbal IQ testing during childhood has revealed considerable change within individuals, reflecting different rates of developmental maturation, and, specifically, language development (Breslau et al. 2001).

## Results

### Attachment by the MCAST and the SBST

The distribution of four-way attachment classifications according to the MCAST was 73.5 % *secure (B)* (*n* = 86), 9.4 % *avoidant (A)* (*n* = 11), 15.4 % *ambivalent (C)* (*n* = 18) and 1.7 % *disorganized (D)* (*n* = 2) with respect to mother, and 68.3 % *secure (B)* (*n* = 82), 16.7 % *avoidant (A)* (*n* = 20), 10 % *ambivalent (C) (n* = 12) and 5 % *disorganized (D)* (*n* = 6) with respect to father. Our distribution concerning father attachment is consistent with other studies (Lucassen et al. 2011). For statistical power, further analysis was based on a Secure/Insecure classification from MCAST. No gender differences were found with respect to attachment to mother (*χ* ^*2*^_*(1)*_ = .003, *p* > .05) or father (*χ* ^***2***^_*(1)*_ = .26, *p* > .05), nor were there any effects of child age (entered in a logistic regression with dichotomous attachment classification as outcome variable) for mother *B* = .02, *p* = .67 and father *B* = −.03, *p* = .49. However, a difference was found in verbal ability of children with secure, compared to children with insecure, attachment classification with respect to mother (*F*_*(1, 115)*_ = 6.40, *p* < .05, *η* ^*2*^ = .001), and insecurely attached children scored significantly lower than securely attached (Secure: *M* = 92.33, SD = 1.62; Insecure: *M* = 84.23, SD = 2.76), logistic regression *B* = −1.01, *p* < .0001. Thus verbal IQ (T1, measured concurrently with the MCAST) was entered as covariate in further analysis regarding MCAST attachment to mother. There was no difference in verbal ability between children with secure vs. children with insecure attachment to father (*F*_*(1, 118)*_ = .14, *p* > .05, *ns*).

The mean of the secure base script (SBS) compound for mother (*M* = 5.56, SD = 1.63) and for father (*M* = 5.46, SD = 1.63) based on the MCAST assessments did not differ (*t* = −.85, *ns*). No gender difference was found with respect to SBS/MCAST for mother or father. Verbal IQ at test-session 1 was unrelated to the father-SBS compound (*r* = .07, *ns*) but, as expected, related to the mother-SBS compound (*r* = .28, *p* < .001).

Preliminary analysis was conducted to assess possible influences of verbal fluency, participants’ age and gender on SBST scores. Similarly to previous findings (Psouni and Apetroaia 2014; Psouni et al. 2015), parent-child and friend-child story sub-scores were highly correlated (*r* = .77, *p* < .001). Total SBST scores ranged between 1.13 and 6.50, were independent of child’s age (*r* = .18, *p* > .05) and gender (*r*_*s*_ = −.03, *p* > .05) but correlated with verbal IQ at test session 3 (*r* = .46, *p* < .001).

Data were dichotomized according to a cut-off score of 4, defined as unequivocally demonstrating the presence of a secure base script (Psouni and Apetroaia 2012), which is also supported by previous data (Psouni and Apetroaia 2014). Thus, 32 children (59.3 % girls) who demonstrated no scripted secure base knowledge in their stories were categorized in a low scriptedness, “insecure” group, and 43 children (55.8 % girls) were categorized in a high scriptedness*,* “secure” group, resulting in a distribution consistent with other data (Cassibba et al. 2013; Psouni and Apetroaia 2014; Van IJzendoorn and Bakermans-Kranenburg 1996).

### Relationship between Attachment Representations to Mother and Father, based on the MCAST

Concordance between attachment to mother and father was assessed for 117 children. Significant concordance was found for the two-way classification (*Cohen’s k* = .32, *p* < .0001), with 58.1 % of children (*n* = 68) showing representations of secure attachment to both parents (compared to 59 % concordance in Fox et al. 1991; 62 % concordance in Van IJzendoorn and De Wolff 1997; 65 % in Diener et al. 2008) and 14.5 % (*n* = 17) showing representations of insecure attachment to both parents. Thus, for just over 72 % of children, the 2-way attachment classification with respect to one parent predicted the 2-way attachment classification with respect to the other parent (Table 1). Turning to continuous measures, the MCAST-SBS compounds for mother and father did not differ in magnitude (Mmother = 5.46, SD = 1.43 Mfather = 5.58, SD = 1.63) and were highly correlated (*r* = .55, *p* < .0001) after controlling for verbal IQ (since it was significantly related to the mother-SBS compound). Even not controlling for verbal IQ, the correlation between the mother MCAST and father MCAST SBS compounds was *r* = .55.

**Table 1:** Concordance attachment to mother and attachment to father (two-way classifications, MCAST) (*N* = 117)

	Secure_mother_	Insecure_mother_	Total
	*N* (%)	*N* (%)	*N* (%)
Secure _father_ *N* (%)	68 (58.1 %)	13 (11.1 %)	81 (69.2 %)
Insecure _father_ *N* (%)	19 (16.3 %)	17 (14.5 %)	36 (30.8 %)
Total *N* (%)	87 (74.4 %)	30 (25.6 %)	117 (100 %)

*Note* Pearson’s *χ* ^*2*^_*(1, 117)*_ = .32, *p* = .001. The prediction of father MCAST two-way classification (secure vs. insecure) from mother MCAST classification is 72.6 %

### Relationship between MCAST and SBST

As verbal IQ measurements between first and third test sessions (about 1 year interval) were highly associated (*r*_(75)_ = .60, *p* < .01), similarly to other findings (Neyens and Aldenkamp 1997), and MCAST-SBS mother compound and SBST scores correlated with verbal IQ, verbal IQ was controlled for in subsequent analyses by the third test-session scores. There was no categorical convergence between 2-way attachment classification with respect to mother (MCAST: Secure vs. Insecure) and scripted secure base knowledge (SBST: high vs. low scriptedness) (*χ* ^*2*^_*(1)*_ = .94, *p* = .33, *ns*). However, significant convergence was found between 2-way attachment classification with respect to father (MCAST) and SBST (*χ* ^*2*^_*(1)*_ = 3.67, *p* < .05, *Cohen’s k* = .19, Table 2). Consistent with that, children securely attached to father (MCAST) received significantly higher scores in SBST about a year later (*F*_*(1,72)*_ = 3.96, *p* < .05, *η* ^*2*^ = .05), controlling for verbal IQ (since SBST-scores were related to verbal IQ). Turning to continuous variables, convergence between the MCAST SBS compound and SBST scriptedness scores was found for father (*r* = .25, *p* < .05) but not mother (*r* = .16, *p* > .05), controlling for verbal IQ. The pattern was the same for the SBST parent-child sub-score only (for MCAST SBS father *r* = .23, *p* < .05, for mother (*r* = .16, *p* > .05).

**Table 2:** Cross tabulation of children’s 2-way attachment classifications based on the MCAST (Secure vs*.* insecure for mother and father, respectively) and the SBST (High scriptedness/secure vs. low scriptedness/insecure) (*N* = 75)

MCAST SBST	Secure_mother_	Insecure_mother_	Total	Secure_father_	Insecure_father_	Total
High scriptedness (S*ecure*)	35	8	43	32	11	43
Low scriptedness (*Insecure*)	23	9	32	17	15	32
Total	58	17	75	49	26	75

*Note* MCAST Mother—SBST, Pearson’s *χ* ^*2*^ (1, 75) = *.*94, *p* = .33, Kappa = −.08; MCAST Father—SBST Pearson’s *χ* ^*2*^ (1, 75) = 3.67, *p* < .05, Kappa = .19. The prediction of SBST classification (high vs. low scriptedness) from MCAST-Father two-way classification is 63 %

## Discussion

The present study provides evidence of concordance in quality of attachment representations to mother and father in 6-to-7-year olds. It also provides, to our knowledge for the first time, some evidence of convergence between two narrative-based attachment assessment methods, conceptually consistent with the presence of scripted secure base knowledge also during the time around transition to middle childhood. Importantly, partial convergence of attachment representations using the two narrative-based methods could be shown; categorical analysis indicated concordance between attachment to father assessed by the MCAST and secure base scripts assessed by the SBST, and continuous score analysis indicated association between secure base scripts with respect to father as assessed by the MCAST and secure base scripts assessed by SBST. Unexpectedly, these findings were not replicated concerning attachment to mother.

Besides the fundamental notion that children maintain separate representations of attachment to mother and father in the first years of life (Belsky and Rovine 1988), it has been argued that relationship-specific representations merge into a unitary pattern by late middle childhood (Dykas et al. 2006), as executive functioning becomes more efficient, allowing better voluntary control of attentional processes, and sophisticated appraisal skills that enable children to integrate multiple and different representations into more abstract models (Zimmermann and Iwanski 2015). Consistent with studies with infants and children older than 8 years (Diener et al. 2008; Fox et al. 1991; Monteiro et al. 2008; Steele et al. 1996; Van IJzendoorn and De Wolff 1997; Veríssimo et al. 2011), our results provide evidence of concordance between attachment representations with respect to mother and father also at age 6-to-7-years. We found specific evidence that mother-related and father-related scripted secure base knowledge, captured by the MCAST SBS compounds, are highly related to one another and, averaged, to the mother-and-father-related scripted secure base knowledge (SBST sub-score). This concordance may result from mother and father within a family behaving similarly in attachment-related situations, providing a coherent familiar environment which results in similar experiences of attachment security in relation to each of the two parents (Fox et al. 1991). Alternatively, there could be an influence of the mother’s attachment state of mind on the father-child attachment relationship (Allen and Hawkins 1999; Steele et al. 2008). This account, also referred to as “maternal gatekeeping” (Allen and Hawkins 1999), assumes that maternal state of mind with respect to attachment impacts on features of the father’s relationship to the child, for example, frequency and context, and on the child’s behaviors in interactions beyond the child-mother relationship. Recent findings that father unavailability may impact on the child’s attachment security through influences on the mother’s ability to provide sensitive caregiving to the child (Booth-LaForce et al. 2014) point to influences also in the opposite direction.The convergence of scripted knowledge of attachment security found is consistent with other studies assessing attachment through story completion tasks concurrently (Bretherton et al. 1990; Cassidy 1988; Psouni and Apetroaia 2014) and over time (Ammaniti et al. 2005; König et al. 2007).

An alternative explanation for the convergence of MCAST classifications of representations of attachment to mother and father, respectively, may be sought in the MCAST coding system, as also discussed by Barone et al. (2009). The categorical attachment classification of each vignette is done using a prototype-based coding system that considers content as it is reflected in the behaviors of child and parent dolls in the child’s narrative, and other features of the child’s representation reflected in the narrative, such as coherence and mentalization. Thus, the four assessed areas by the MCAST, all contributing per se to the definition of the overall attachment classification, may not be independent from one another. Furthermore, if coherence and mentalization as reflected in the child’s narratives are relatively stable characteristics of the child, they are likely to be expressed similarly across narratives, resulting in common influence of the separate MCAST classifications of attachment to mother and father, respectively. MCAST procedures for separate assessments of attachment to mother and father have previously been presented (Barone et al 2009; Barone and Lionetti 2011) but no evidence of construct validity was discussed in these studies. Notably, no study using the MCAST has undertaken an empirical scrutiny of the MCAST coding system to this end.

The lack of association between scripted knowledge of secure base with respect to mother as reflected in MCAST and SBST (about 1 year later) is not consistent with evidence of stability in attachment security to mother assessed longitudinally using observational measures (e.g., Moss et al. 2005), representational measures (Gloger-Tippelt et al. 2002; Green et al. 2000; Seven and Ogelman 2013; Toth et al. 2000), or both (Bureau and Moss 2010). While the correlation between attachment security to mother (both by the MCAST and by the SBST) and verbal ability is not surprising (Stievenart et al. 2010, 2014; van IJzendoorn et al. 1995; van IJzendoorn and Van Vliet-Visser 1988), in assessing the relation between attachment security by MCAST-mother and SBST, there is a risk that the two measures’ concordance is confounded by the shared variance with verbal abilities, and becomes non-significant when verbal ability is controlled for. (Indeed, the correlation between scripted knowledge of secure base with respect to mother in MCAST and SBST was .30, significant at *p*<.01 before controlling for verbal IQ). On the other hand, consistent with other findings (Verschueren and Marcoen 1999), our assessments of attachment to father were unrelated to verbal ability and associations between these assessments were unaffected when controlling for verbal IQ. Indeed, meta-analytic evidence suggests that, compared to mothers, fathers are overall less verbal, and use less supportive, less negative, more directive and more informative language when interacting with their children (Leaper et al. 1998; Tamis-LeMonda et al. 2004). Therefore, the socioemotional context evoked by the MCAST with respect to mother and father, respectively, may have elicited different linguistic skills, depicting these differences in the linguistic context of the relationship to each parent.

Children’s scripted knowledge of attachment in the SBST was associated with earlier attachment scriptedness specifically when testing attachment to father with the MCAST, supporting the notion that features in the early relationship to father in particular contribute to the child’s scripted secure base knowledge (Steele et al. 2014). Also using a categorical analytical approach, convergence of secure base knowledge assessed by MCAST and SBST was found for father but not mother. Possibly, this reflects a distinct change of importance of the father as parent, especially concerning the support of exploration (Bretherton 2010; Paquette 2004). Alternatively, MCAST assessments of attachment to mother and father, separately, may be subject to cultural bias: mother figures placed in the kitchen cooking (as prompted by the interviewer in “hurt knee” and “tummy ache”) is entirely consistent with Italian cultural expectations and, very likely, also with the children’s experiences. By contrast, when the assessment concerned father in the present study, father in the kitchen cooking was perceived by many children as unusual and peculiar. In several cases, children discounted this framework as implausible, and specific elaboration was necessary: agreeing with the child that it is Sunday made it plausible to place the father in the kitchen, agreeing that the mother was at the hospital with grandmother made it plausible to have the father soothe after nightmares, agreeing that father and child were out shopping alone because they would buy a present for the mother to surprise her made plausible the shopping-center vignette.

The novelty of a father figure who is standing in the kitchen elicits, perhaps, stories with much attention around this father, rendering narratives more elaborate. More importantly, we suggest that the MCAST-mother and MCAST-father may not be capturing the mother and father versions of the same construct. When children make narratives according to the MCAST vignettes and a mother doll, their narrative may be the direct reflection of their experience-based attachment scripts. By contrast, when making narratives to the same MCAST vignettes but with a father doll, it appears that children apply their knowledge and expectations in familiar attachment-related situations to someone who does not *usually* exist in these situations with the child. This can be seen as a step toward a generalization of relation-specific attachment representations. Thus, MCAST-father may already be capturing a degree of generalization in the child’s attachment representations, more likely to be associated with the later measure of the child’s secure base scripts (SBST) as it reflects a more general rather than relationship-specific representational level (Bretherton and Munholland 2016; Psouni and Apetroaia 2014). In that sense, the present findings are more consistent with a generalization process of attachment from mother (as primary caregiver) to father, rather than with the idea that convergence reflects similarities between caregivers in the family environment in answering the child’s attachment needs.

Our findings highlight the methodological challenge related to the assessment of attachment to father in middle childhood. It has already been suggested that the importance of the attachment relationship to father may have been underestimated as a result of a tendency of attachment measures to focus on the role of attachment figures in safe haven, rather than secure base, situations (Kerns et al. 2015). Given that the father’s role has been recognized as central for supporting the child’s exploration (Bretherton 2010; Di Folco and Zavattini 2014; Grossmann et al. 2008), this tendency may be seen as a bias when father-child attachment is considered. Indeed, Kerns et al. (2015) found that most children reported secure base and safe haven support from both parents, but were also shown to prefer mother for safe haven and father for secure base support. The present findings suggest the additional complication of using contexts and vignettes that may be cultural and relationship-specific, for the measurement of attachment across relationships (and cultures). Further exploratory research may be necessary to clarify whether attachment to father in middle childhood is experienced in different contexts, and differently, as compared to attachment to mothers, and to also determine the premises for such differences. Where parental roles are still highly gender diversified, it is perhaps necessary that measures encompass this diversification, but in contexts where parents are equally engaged in parental caregiving also during the child’s infancy this diversification may not exist. Importantly, all attachment measures share unique variance, as they are all based on the connection with the underlined secure base script functioning, but different methods may vary on the basis of whether they consider specific relationships or more general characteristics of the child (Kerns et al. 2000; Psouni and Apetroaia 2014), so the fact that two measures do not correlate highly does not mean one of the two is flawed or not attachment related (Bosmans and Kerns 2015).

### Limitations

There are limitations in the present study. First, notwithstanding the high rate of consent to participation (78 %), participation was voluntary and based on parents’ consent, so a self-selection bias cannot be excluded. Despite the limited number of families not willing to continue participating (22 of 120 families, approximately 18 %), attrition also poses a limitation to the generalizability of the findings. Second, the sample size did not allow discrimination between different insecure attachment patterns (ambivalent, preoccupied, or disorganized), nor was it possible to keep track of attachment disorganization in specific with respect to the study questions. Third, negative life events during the 12-month-period of assessments were not addressed and could thus not be accounted for. Thus, the study missed possible changes in features of the caregiving environment, for instance occasional parental illness (Weinfield et al. 2004), that may be of importance for attachment development in low risk contexts. Finally, potential training effects in children’s ability to tell coherent stories based on prompts over the three sessions cannot be excluded. Working with the MCAST at session 1 may have resulted in familiarization with story completion tasks, and with the specific attachment themes the stories build upon. Such training effects may have confounded the measurement of attachment with the MCAST at test-session 2 and the SBST at test session 3.

Despite these limitations, based on two different narrative completion tasks for attachment assessment, the results from the present study provide some evidence of predictive validity of the MCAST and construct validity for both MCAST and SBST. With an interval of about a year at a time during transition to middle childhood, the MCAST and SBST as two different representational measures of attachment, and combining categorical and continuous approaches for attachment assessment, partly converged. The current results suggest that, in the beginning of middle childhood, the secure base script may not be a fixed construct built upon past experiences but rather the result of how easily a child can generalize different scripts from specific interpersonal relationships and situations, to fit new situations and relationships. Such first signs of a process of generalization indicate that the assessment of attachment as a more general, non-relationship-specific, characteristic of the middle-childhood child is meaningful, but the predictive validity of such assessment needs yet to be established. Importantly, patterns of socialization of emotions and conversational style in the family may be influencing both the coherence in children’s narratives by the MCAST (and, consequently, their classifications as secure) and the production of detailed and rich SBS narratives by the SBST. The openness with which a child can retrieve attachment scripts and adapt them to different contexts and people may depend on a combination of level of security, general developmental maturation and social experiences. Further exploration and validation of narrative-based measures of attachment security in childhood is essential for a better understanding of the process of development from relationship-specific attachment representations to generalized attachment scripts.
